# Spatial immune dysregulation in MASLD: integration of lobular zoning, metabolism and immune function

**DOI:** 10.3389/fimmu.2026.1922207

**Published:** 2026-08-28

**Authors:** Dandan Gao, Xiaolei Wang, Shumin Mu, Yong Sun, Zhongming Wu

**Affiliations:** 1 The First Clinical Medical College, Shandong University of Traditional Chinese Medicine, Jinan, China; 2 Key Laboratory of Endocrine Glucose and Lipid Metabolism and Brain Aging, Ministry of Education, Jinan, China; 3 Department of Endocrinology, Shandong Provincial Hospital, Jinan, China; 4 Endocrine and Metabolic Diseases Hospital of Shandong First Medical University, Shandong First Medical University & Shandong Academy of Medical Sciences, Jinan, China; 5 Affiliated Hospital of Shandong University of Traditional Chinese Medicine, Jinan, China

**Keywords:** immune exhaustion, liver lobule partitioning, macrophage heterogeneity, metabolic dysfunction-associated steatotic liver disease, regional exhaustion index, spatial immunology

## Abstract

Most prior studies on immune-related fatty liver disease have focused on changes in immune cell quantity and composition. However, this “cell-count model” does not fully explain the regional onset of fibrosis, spatial heterogeneity of lesions, or variable immunotherapy responses. Immune dysregulation in metabolic dysfunction-associated steatotic liver disease (MASLD) involves not only compositional changes but also spatial redistribution and functional alterations of immune cells within specific hepatic lobule regions. Emerging multi-omics and spatial transcriptomic data suggest that chemokine gradient remodeling, metabolic microenvironment reprogramming, and circadian rhythm disruption may drive immune cell relocation along the portal-central axis. We propose that immune dysregulation in MASLD reflects a disruption of spatial immune homeostasis and introduce the liver lobule as the basic unit for spatial immune analysis. The lobule comprises three functionally distinct zones: the periportal zone (zone 1), which serves as the primary immune surveillance barrier; the pericentral zone (zone 3), characterized by metabolic stress and functional impairment; and the fibrotic septum, which in advanced disease creates a structurally confined immunosuppressive niche. To quantify regional T cell dysfunction, we propose a Regional Exhaustion Index (REI) as a conceptual framework, calculated as the ratio of PD-1^+^TIM-3^+^ CD8^+^ T cells to total CD8^+^ T cells within a defined microanatomical region. This metric translates regional immune conditions into a quantifiable measure of T cell functional impairment across different lobular zones. We outline how chemokine remodeling, metabolic reprogramming, and circadian disruption drive spatially distinct immune alterations: reduced immune surveillance in zone 1, functional impairment in zone 3, and structural confinement in the fibrotic septum. We also discuss the translational potential of this framework for biomarker discovery, patient stratification, and targeted therapy. The REI remains an exploratory metric requiring validation of its biological meaning and clinical utility. Current evidence is largely correlational, and the causal role of spatial immune changes in disease progression demands further investigation. Nonetheless, this spatial perspective complements existing paradigms and may guide the development of spatially targeted therapeutic strategies.

## Graphical Abstract

**Figure:**
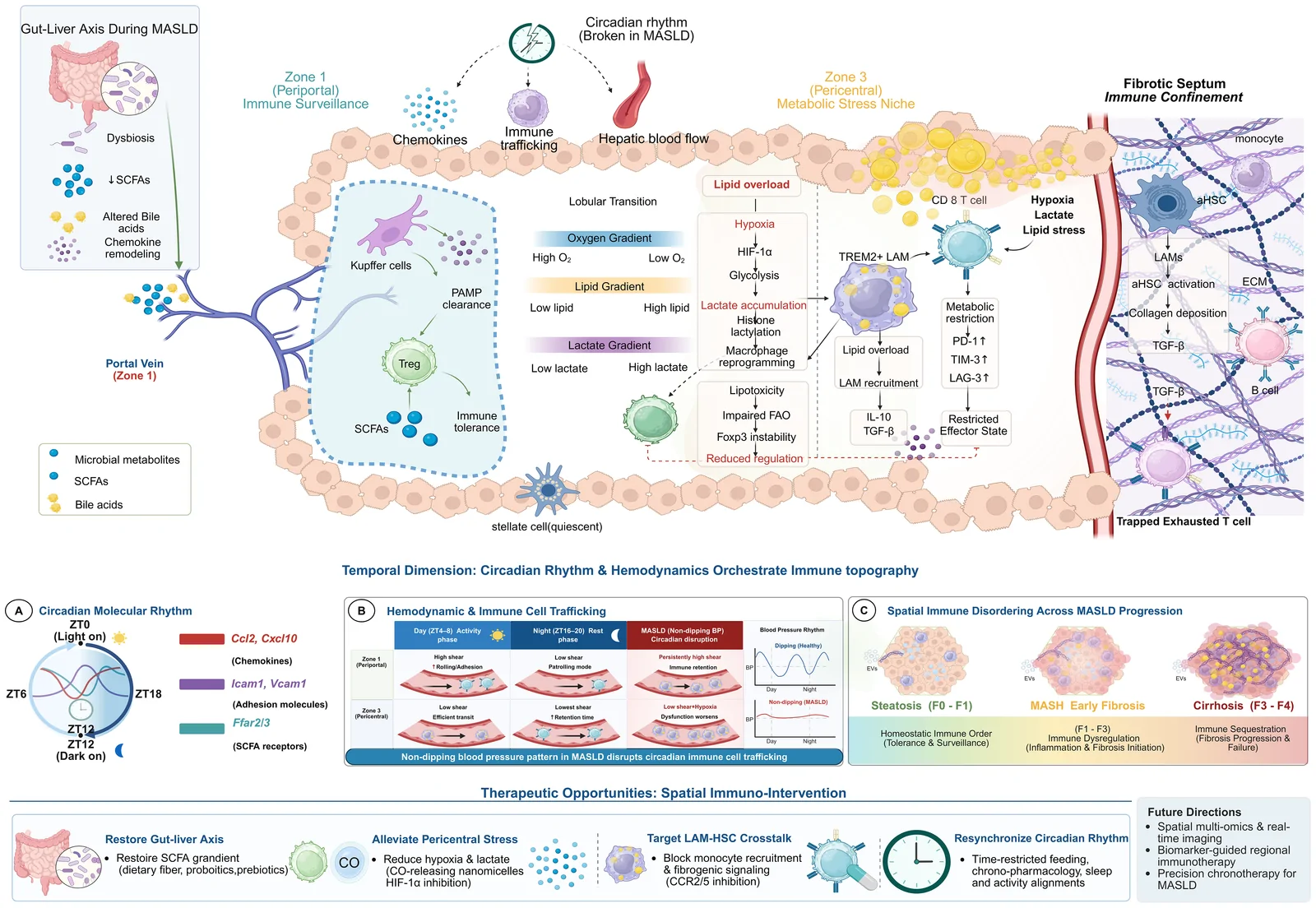
Created in BioRender. Gao, N9. (2026) https://BioRender.com/h17u8au.

## 1 Introduction

Metabolic dysfunction-associated steatotic liver disease (MASLD) affects approximately 30% of the global adult population and is the fastest-growing underlying cause of hepatocellular carcinoma (HCC) (1–3). During its progression from simple steatosis to metabolic dysfunction-associated steatohepatitis (MASH), fibrosis, cirrhosis, and ultimately HCC, abnormal activation of the immune system is believed to contribute to disease progression (1, 4–6). Over the past two decades, research has primarily focused on changes in the quantity and phenotypic polarization of immune cells. For example, the number of immunomodulatory Kupffer cells (KCs) decreases; infiltration of pro-inflammatory Ly6C high-expressing (Ly6C^hi) monocytes increases; embryonically derived KCs are gradually replaced by bone marrow-derived macrophages; CD4^+^ and CD8^+^ T cells accumulate in the liver; and regulatory T cells (Tregs) are relatively reduced (7, 8).

However, as research has progressed, this paradigm has gradually revealed its limitations in explaining several key pathological features of MASLD. First, the inherent metabolic zonation of liver lobules dictates that immune function cannot be uniformly distributed across space. This spatial heterogeneity is a hallmark of liver physiology, as hepatocytes in the periportal area (zone 1) and the pericentral area (zone 3) perform distinct metabolic functions. Hepatocytes in zone 1 preferentially engage in gluconeogenesis and fatty acid β-oxidation, whereas those in zone 3 tend toward glycolysis and lipogenesis (9). This implies that different metabolic zones may require distinct immune regulatory mechanisms, suggesting that an effective immune response must adapt to these highly specialized local microenvironments (10).

Second, fibrosis in MASLD does not occur diffusely. Recent spatial multi-omics studies, using unbiased spatial transcriptomics analysis, have identified a highly specific fibrosis gene program that is spatially enriched in the pericentral area and exhibits peak activity during the late fibrosis stages (F3–F4) (11). This finding suggests regional selectivity in disease progression at the spatial level. Third, although techniques such as single-cell RNA sequencing have revealed the high heterogeneity of immune cells and identified subpopulations such as lipid-associated macrophages (LAMs) that play key roles in MASLD (12), critical spatial positional information is lost during tissue dissociation. The advent of spatial transcriptomics offers a novel technical approach to resolving cell distribution and functional status *in situ* within the tissue (13, 14).

An increasing number of studies are now focusing on the spatial dimension of immune responses. Existing evidence suggests that in MASLD, the spatial distribution patterns of immune cells may change. For instance, the number of LAMs increases significantly with disease severity and specifically accumulates in the central-peripheral regions, closely relating to the local metabolic environment and inflammatory state (11). Moreover, the clinical management of MASLD faces significant bottlenecks that vary by disease stage. In early stages, lifestyle intervention remains the primary approach, but adherence is poor and metabolic comorbidities often persist. In advanced fibrosis and cirrhosis, there are no approved antifibrotic therapies, and disease progression is frequently complicated by portal hypertension and liver failure. For MASLD-associated HCC, immunotherapy has shown suboptimal responses compared to other etiologies, with lower objective response rates and higher rates of hyperprogressive disease, partly attributed to the unique immunosuppressive microenvironment shaped by chronic metabolic inflammation (15). These clinical challenges underscore the urgent need to understand the spatial organization of immune dysfunction, as regional variations may influence both disease progression and therapeutic efficacy. In this context, this paper proposes that immune dysfunction in MASLD may partly reflect a disruption of immune spatial homeostasis, that is, the distribution of immune cells within liver lobules deviates from its normal pattern, exposing them to different metabolic and signaling environments and subsequently affecting their functional output. This spatial perspective does not negate the importance of changes in immune cell numbers; rather, it suggests that understanding where immune cells are located and how their localization affects function is equally critical for comprehending disease pathogenesis.

## 2 Drivers of spatial immune dysregulation

**Figure 1:**
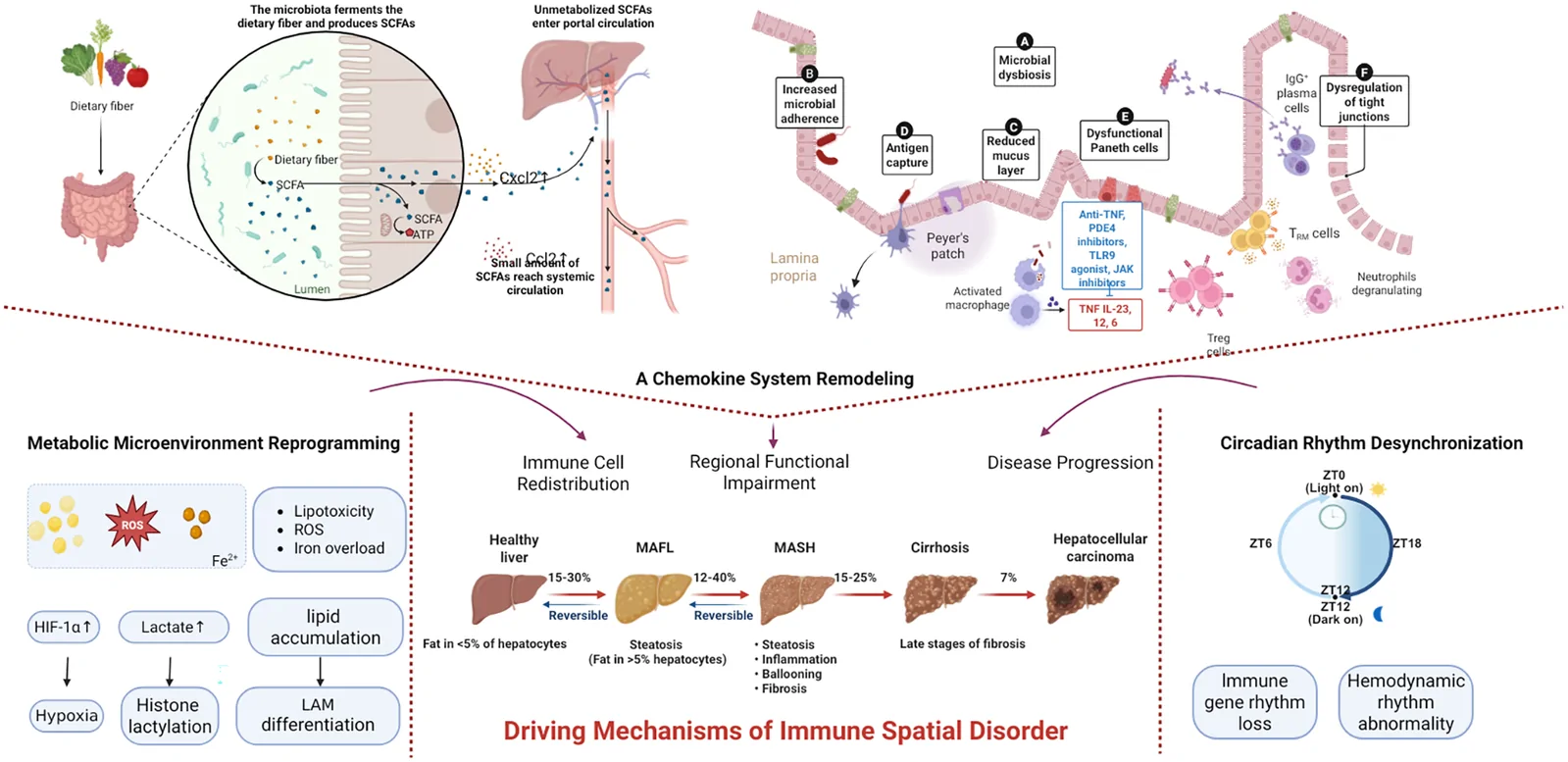
Drivers of spatial immune dysregulation. Created in BioRender. Gao, N9. (2026) https://BioRender.com/w8qfc0d.

Immune cells are not evenly distributed within the hepatic microenvironment; they perform distinct functions and must balance immune tolerance with immune surveillance (16, 17). Based on current research, several mechanisms are believed to underlie the spatial distribution of immune cells, including chemokine gradients, metabolic microenvironmental factors, and circadian rhythms (18, 19). Although these factors operate in harmony under physiological conditions, they may be altered in the context of MASLD, thereby contributing to inflammation and tissue damage. These three categories of drivers, chemokine remodeling, metabolic reprogramming, and circadian disruption are not mutually exclusive but rather converge to reshape the spatial immune landscape (**Figure 1**).

### 2.1 Chemokine system remodeling

The chemokine system regulates the recruitment of immune cells and their distribution within tissues, influencing cell-to-cell interactions (20, 21). In a healthy liver, certain chemokines show regional expression along the portal vein-central vein axis, which helps maintain the relatively stable distribution of immune cells across different microanatomical regions (22).

#### 2.1.1 Gut-liver axis and microbiota-associated immune regulation

The gut microbiome continuously interacts with the host through metabolites and immune signals, playing a crucial role in MASLD-related immune regulation (23, 24). Zhou et al., through multi-kingdom analysis, revealed that in MASLD patients, not only is the bacterial community imbalanced, but the viral and fungal communities also undergo coordinated dysbiosis. Specifically, there is an enrichment of active *Ruminococcus* and a depletion of *Prevotella fecalis* and its associated phages. This cross-kingdom ecological imbalance is closely associated with abnormal bile acid metabolism, suggesting that disruption of the phage-bacteria-metabolite axis may be a key event driving liver immune dysregulation (25). Qiao et al. further discovered that gut-derived tryptophan metabolites, such as 5-hydroxyindoleacetic acid (5-HIAA), can induce downstream fatty acid oxidation by activating the aryl hydrocarbon receptor (AhR) in the gut, thereby exerting a protective effect on MASLD. This finding suggests that specific microbial-derived metabolites can regulate the hepatic inflammatory environment (26). Additionally, the concept of the “oral-gut-liver axis” has been proposed in chronic liver diseases. Dysbiosis of the oral microbiota and its associated virulence factors may exacerbate the immune burden on the liver through translocation across the intestinal barrier (27). Critically, these microbiota-derived metabolites and signals directly influence hepatic chemokine production. For instance, microbial metabolites such as short-chain fatty acids (SCFAs) and secondary bile acids have been shown to modulate the expression of CCL2, CXCL10, and other chemokines in hepatocytes and liver sinusoidal endothelial cells via activation of specific receptors (28). Dysbiosis-associated reduction in SCFAs and alterations in bile acid composition can thus reshape the chemokine gradient along the portal-central axis, potentially altering the recruitment and positioning of immune cells. Furthermore, microbial translocation and PAMP signaling can induce KC activation, leading to increased secretion of chemokines such as CXCL1 and CXCL2, which may further disrupt the normal chemokine gradient. Collectively, these findings suggest that remote signals derived from the microbiota may reshape the inflammatory factor spectrum both systemically and locally, potentially contributing to abnormalities in the chemokine gradient within the liver. This imbalance may play a role in the spatial immune dysregulation observed in MASLD.

#### 2.1.2 Changes in chemokine expression and abnormal immune cell distribution

Under the continuous influence of intestinal signals and metabolic stress, the expression patterns of liver chemokines in MASLD may change (29, 30). Relevant studies have shown that in end-stage liver diseases such as acute-on-chronic liver failure (ACLF), the recruitment and spatial distribution of immune cells are significantly reshaped by the metabolic microenvironment and inflammatory signals. This process is manifested by impaired phagocytic function of innate immune cells, excessive formation of neutrophil extracellular traps, and dysfunction of monocytes/macrophages, which may contribute to impaired pathogen clearance and uncontrolled local inflammation (31). Although MASLD is less severe than ACLF, similar molecular pathways are activated (32). Additionally, Tregs, which are key guardians of immune balance, are also affected by the microenvironment. In the tumor context, alterations in Treg function may influence the immunosuppressive state (33). These studies suggest that changes in the immune microenvironment may affect both the distribution and functional status of immune cells.

### 2.2 Metabolic microenvironment remodeling

Compared to the regulation of immune cell migration pathways by chemokines, the liver’s inherent metabolic compartments and their remodeling under pathological conditions primarily influence the functional state, differentiation trajectory, and spatial distribution patterns of immune cells by altering local microenvironmental characteristics (34). Metabolic signals can change the characteristics of immune cells and establish specialized immune niches through regional differences, thereby contributing to the spatial organization of the immune system in the liver (13, 35, 36).

#### 2.2.1 Hepatocyte metabolic stress and immune microenvironment signals

As the primary metabolic cells of the liver, hepatocytes under stress directly affect the composition of the local immune microenvironment (37). Lipid toxicity, iron homeostasis dysfunction, and oxidative stress in MASLD all alter the release pattern of hepatocytes and thus influence the recruitment and activity of nearby immune cells. Based on a study of a non-alcoholic fatty liver disease model, iron overload increases expression of fetuin-A through activation of FoxO1, and a similar mechanism may also occur in the liver in MASLD (38). Hepatoma-derived growth factor (HDGF) has also been found to promote the STAT3 pathway for lipid synthesis and modulate macrophage function via exosomes (39). Although most of the above evidence originates from studies on tumors, it is plausible that secreted factors from hepatocytes may also alter the behavior of immune cells. In addition, the transcription factor TCF19 is thought to regulate the response of hepatocytes to lipid toxicity, and a reduction in its expression has been linked to inflammation and increased fibrosis (40). Hepatocytes under metabolic stress release signaling molecules into the local environment, creating concentration gradients of signals in specific regions of the liver lobule and thus potentially altering the distribution and function of immune cells.

#### 2.2.2 Hypoxic microenvironment and the HIF-1α signaling axis

With the progression from MASLD to MASH, abnormal lipid accumulation in hepatocytes increases, leading to cell enlargement. Fibrosis in the perisinusoidal space and activation of hepatic stellate cells can also cause a narrowing or even occlusion of hepatic sinusoids (41). These physical changes increase resistance to portal blood flow and decrease the rate of oxygen diffusion from the sinusoidal lumen to the liver tissue, thereby creating a localized hypoxic microenvironment within the liver lobules. This phenomenon is especially prominent in zone 3, which already has a relatively low oxygen level, establishing a cycle of establishing “hypoxia-fatty degeneration-hypoxia”. This cycle may serve as a spatial driver for the development of metabolic disorders, inflammatory necrosis, and fibrosis in the disease (42).

HIF-1α is a central transcription factor that regulates genes in response to hypoxia, enabling cellular adaptation to low-oxygen environments (43). Recent research has suggested that HIF-1α may act as an “early promoter” of MASLD development. In a mouse model of MASH induced by a high-fat diet and choline-methionine deficiency, HIF-1α was significantly increased before the development of serious histological injury and inflammation. Nano-gels with carbon monoxide-donating groups have been shown to inhibit HIF-1α functionally, thereby improving metabolic syndrome and reducing the activation of the NLRP3 inflammasome and liver fibrosis. Based on these data, HIF-1α may be involved in the signaling pathways of inflammation and fibrosis caused by metabolic stress (44).

At the same time, another HIF-1α regulatory signal, IL-33 released by the intestine, may further promote MASLD via two pathways. First, IL-33 in intestinal epithelial cells binds to HIF-1α and suppresses the expression of genes for intestinal barrier repair. Secondly, IL-33 released in the lamina propria can activate HIF-1α in CD4^+^ T cells via the ST2 receptor, promoting Th1 differentiation and disturbing the intestinal immune system, which may cause liver damage through the bacterial metabolite trimethylamine oxide (30).

Obstructive sleep apnea-induced chronic intermittent hypoxia is an independent risk factor for the development of MASLD and has a different mechanism of pathogenesis than persistent hypoxia (42). Transcriptome analysis has identified a specific effector, KLF9, which directly binds to the GC-rich motif in the promoter region of *Nr4a1* and represses *Nr4a1* transcription. Downregulation of p38 MAPK phosphorylation occurs, releasing inhibition of gene expression for lipid synthesis and inducing fatty liver disease and inflammation in hepatocytes. KLF9 induction does not require the conventional HIF-1α pathway, suggesting that a separate oxygen-sensing signal transmission route for intermittent hypoxia may exist (45). CX3CL1 from hepatic sinusoidal endothelial cells is also increased after episodes of hypoxemia. It acts on the CX3CR1 receptor of KCs to activate CaMKIIδ signaling and promote nuclear translocation of HDAC4, thereby releasing transcriptional repression of *Rubicon* and inhibiting KC autophagy while promoting apoptosis. Consequently, the immune defense and damage control functions of the liver may be compromised, and the progression of MASLD may be further accelerated (46).

#### 2.2.3 Lactic acid metabolism and epigenetic reprogramming of macrophages

In the hypoxic microenvironment, the glycolytic pathway in liver cells and immune cells is highly active, producing large amounts of lactate at that site (47, 48). Lactate has traditionally been regarded as the final product of anaerobic metabolism, but more recently, it has also been identified as a signaling molecule and an epigenetic regulator. As MASLD progresses, lactate produced during energy metabolism may affect the behavior of immune cells by modulating histone lactylation at a post-translational level (49).

Lactic acid-induced histone lactylation represents a mechanism connecting metabolism and epigenetics (50). In patients with MASLD and corresponding animal models, liver macrophages show an increase in HK2, a typical glycolytic enzyme, and a significant rise in histone H3K18 lactylation (51). This modification promotes the transcription of glycolysis-related genes as well as the expression of pro-inflammatory phenotype in macrophages. Functionally, inhibition of HIF-1α or specific knockout of HK2 in myeloid cells have been shown to reduce lactate production and histone lactylation to a certain extent, thereby alleviating liver inflammation and fibrosis (29, 52). These findings suggest that the metabolic gradient along the “hypoxia-glycolysis-lactate-histone lactylation” axis may alter macrophage behavior via epigenetics and may amplify the local inflammatory response in zone 3.

Lactate accumulation under hypoxia and the phenotypic changes of macrophages in metabolic reprogramming are closely related and regulated bidirectionally (53). Single-cell transcriptomics have revealed that during progression from MASLD to MASH, LAMs and the DTNA^+^ macrophage subpopulation are significantly expanded. These cells show increased glycolytic activity, M2-like polarization characteristics, and activation of the hypoxia/HIF signaling pathway (41). Notably, immune metabolic reprogramming mediated by β-arrestin 2 offers a new perspective on this process. Research has indicated that the absence of myeloid β-arrestin 2 suppresses the differentiation of M1-type macrophages and slows the advancement of MASH by promoting IRG1-mediated succinate production, inhibiting succinate dehydrogenase activity, and reducing the release of mitochondrial reactive oxygen species (54). Succinate is a metabolic intermediate of the tricarboxylic acid cycle that inhibits inflammation and differs from lactate, suggesting that these two metabolites may have different or opposing effects on macrophage polarization (55). The nitrate-nitrite-NO pathway may also modulate the polarization and function of CD206^+^/CD11c^+^ macrophages by altering sialin-mediated macrophage rebalancing to treat metabolic syndrome (56).

Hypoxia and lactic acid metabolism may also alter T-cell function. Succinate may increase the expression of exhaustion markers in CD8^+^ T cells via epigenetic modification in MASLD-associated HCC and thus inhibit their anti-tumor activity (57). Oxidative lipid uptake by CD36 may cause lipid peroxidation and subsequent dysfunction of CD8^+^ T cells (58). Based on the above analysis, accumulated metabolites in the hypoxic area affect the body’s natural defense system and alter the immune landscape under MASLD by regulating adaptive immune responses (59).

### 2.3 Circadian rhythm modulation

The distribution of immune cells in the liver is not static but changes over time in a cyclical manner. Temporal variations in the rhythm of immune-related molecules and alterations in liver hemodynamics may modify the attraction, adhesion, and residence time of immune cells in the liver. Therefore, this regulation may contribute to maintaining and reshaping the liver’s immune spatial organization (60).

#### 2.3.1 Desynchronization between liver molecular clocks and immune gene rhythms

The liver is a peripheral clock organizer regulated by the core clock-gene network (61). These molecular clocks regulate the transcription of genes encoding carbohydrate-and lipid-metabolism enzymes and are involved in the circadian rhythm of some immune-related genes. Research has shown that approximately 15% of the transcriptome in the mouse liver exhibits circadian rhythmicity, and among these, *Ccl2*, *Cxcl10*, and *Icam1* are required components for attracting and binding immune cells (62). When clock gene function is impaired, the rhythmic expression of these molecules is diminished or lost, accompanied by alterations in the composition of liver immune cells (63, 64).

In the context of MASLD, the coupling between molecular clocks and immune regulation may be disrupted. Epidemiological and clinical studies have demonstrated that social time differences, shift work, and similar factors are associated with an increased risk of MASLD (65, 66). A human study further observed that MASLD patients exhibit more pronounced insulin resistance and activation of inflammatory pathways at night, suggesting that the disease may have circadian rhythm-related characteristics (67). Additionally, fibroblast growth factor 1 (FGF1) regulates the circadian rhythm of liver triglyceride secretion, and abnormal expression of FGF1 can exacerbate the diet-induced MASLD phenotype (68).

Time-restricted feeding (TRF) can restore the rhythmic expression of metabolic and inflammatory genes in the liver and improve disease phenotypes by re-establishing the feeding-fasting cycle (69). These findings suggest that disruptions in molecular rhythms may influence the recruitment timing and spatial distribution of immune cells in the liver by altering the temporal expression patterns of immune-related signaling molecules.

#### 2.3.2 Hemodynamic influences on immune cell spatial behavior

In addition to the intracellular molecular clock, the circadian rhythm influences the liver immune microenvironment through systemic physiological parameters. Among these, the circadian variation in blood pressure is particularly significant (70). Healthy individuals typically exhibit a “dipper” pattern, characterized by a decrease in blood pressure at night, which is regulated by the autonomic nervous system and can affect blood flow within the hepatic sinusoids. The rhythm in patients with MASLD is often irregular. Clinical research has shown that elevated blood pressure at night and a non-dipper blood pressure pattern are more prevalent among individuals with MASLD and are positively associated with increased liver stiffness (71, 72). In addition, epidemiological studies have shown that an irregular pattern of blood pressure during sleep and sleep disorders are associated with an increased risk of fatty liver disease and fibrosis, even after adjusting for body mass index (73).

Hemodynamic alterations may modify the location of immune cells in the hepatic sinusoids at the mechanistic level (74). Changes in blood-flow shear stress affect the adhesion of immune cells to endothelial cells in the liver sinusoids, thus altering the distribution and retention of these immune cells. Chronic nocturnal hypertension may change the interface between immune cells and the vascular wall by maintaining a high-shear stress state, a phenomenon that may be more pronounced in the vicinity of the portal vein (75). Further research has shown that mechanical force signals can be converted into biochemical signals by mechanosensitive channels in endothelial cells. Sustained mechanical stress can induce the release of chemokines and adhesion molecules by endothelial cells (76). In this way, the normal rhythmic expression of these molecules may be disrupted, the time course of immune cell recruitment may be modified, and a prolonged, non-periodic accumulation of immune cells in that area may occur. Based on the above mechanisms, interventions that affect the circadian rhythm have been proposed. Adjusting the timing of blood pressure-lowering medications for nighttime use may be suitable for patients with non-dipper hypertension (77). Direct evidence of MASLD is not currently available; therefore, such interventions may alter the liver immune microenvironment indirectly through regulation of hemodynamic rhythm.

Gut dysbiosis in MASLD alters the production of microbial metabolites, including bile acids and tryptophan catabolites, which in turn affect hepatic chemokine expression patterns along the portal-central axis. Local metabolic stress, particularly hypoxia and lipid accumulation, activates HIF-1α signaling and promotes lactate production, leading to histone lactylation in macrophages and supporting the expansion of LAMs and DTNA^+^ macrophage subpopulations. Circadian rhythm disruption, involving both molecular clock desynchronization and abnormal hemodynamic patterns, may further modify the temporal expression of adhesion molecules and chemokines. These three parallel pathways converge to drive the redistribution of immune cells within the liver lobule and to alter their functional states in a region-dependent manner. The convergence of these pathways leads to zone-specific outcomes: reduced immune surveillance in zone 1, functional impairment and chronic inflammation in zone 3, and structural immune confinement in the fibrotic septum.

## 3 The spatial immune landscape of the liver lobule

**Figure 2:**
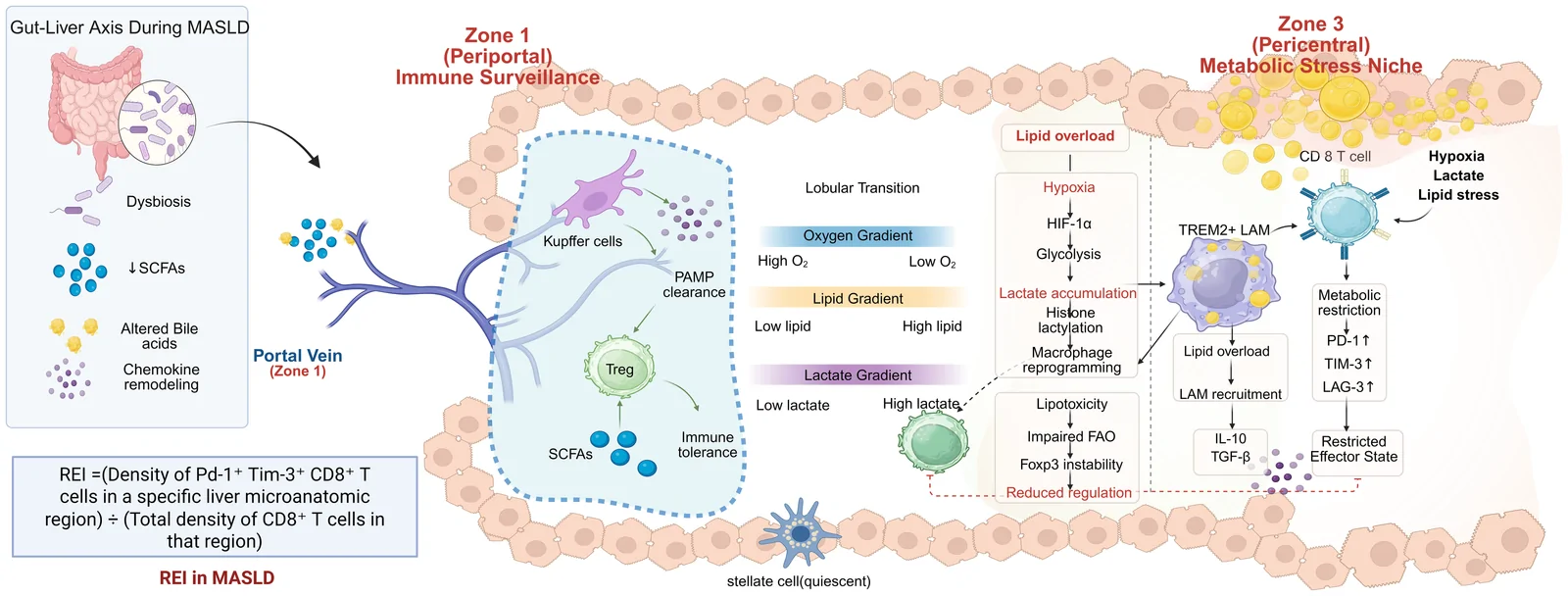
Spatial metabolic-immune landscape of the MASLD liver lobule. Created in BioRender. Gao, N9. (2026) https://BioRender.com/k14y7ez.

The multiple regulators of chemokines, metabolic signals and circadian rhythms collectively establish a spatial gradient in immune function within the liver lobules (78). Concentric zones of high and low metabolic activity and blood flow have formed in the liver lobules; therefore, these areas show structural polarity and, as a result, provide different conditions for the development, activation, and function of immune cells (34). Importantly, the three-zone model presented here is intended as a heuristic framework to organize current evidence, rather than a rigid anatomical division, as the boundaries between zones are gradual and may shift with disease progression. Under conditions of MASLD, the existing partition does not disappear; rather, it is modified under the influence of metabolic stress and inflammatory signals to form areas with distinct immune environments. We systematically characterize the immune landscape of three key spatial compartments, zone 1, zone 3, and the fibrotic septum, at the same time, we describe the spatial differentiation of immune cells, with a particular focus on macrophage heterogeneity and T cell regional exhaustion. We also introduce the Regional Exhaustion Index (REI) as a conceptual framework to quantify regional T cell dysfunction (**Figure 2**).

### 3.1 Zonal organization of immune function

#### 3.1.1 Periportal zone (Zone 1)

The periportal zone (Zone 1) is the primary area for nutrient supply to the liver from the intestine, receiving microbial metabolites and pathogen-associated molecular patterns (PAMPs) (34). In a healthy organism, this region is relatively abundant in KCs and Ly6C^lo-expressing patrolling monocytes that constitute a functional innate immune barrier highly responsive to signals such as endotoxins (79). At the beginning of MASLD, this region shows an increase in the expression of inflammation-related genes. Single-cell transcriptomics have revealed increased chemokines, such as Ccl2 and Cxcl2, and a change in macrophages toward an inflammatory phenotype (80). It should be noted that the existing evidence primarily demonstrates differences in expression among regions; direct longitudinal tracking of dynamic changes in inflammatory signals along the portal vein–central vein axis during disease progression is still unavailable. Therefore, a more cautious interpretation is that as the disease progresses, inflammatory and damaging signals in zone 3 gradually increase, contributing to a change in the overall spatial distribution pattern (81).

#### 3.1.2 Pericentral zone (Zone 3)

The pericentral zone (zone 3) has a relatively metabolically disadvantaged environment that is chronically hypoxic and characterized by high fatty acid and high lactate concentrations (34). These metabolic conditions constitute a constant stress environment that may limit the energy supply and function of immune cells. With the progress of MASLD, accumulating evidence suggests that this area develops an immune reprogramming state due to metabolic stress, not a simple suppression of the immune system, but rather a shift in the activation–limitation balance of immune cell functions (82). Macrophages are also changing significantly at this time. The concentration of TREM2-positive LAMs gradually increases in zone 3. Lineage studies indicate that these cells are mainly derived from peripheral monocytes and develop this phenotype under the influence of local lipid overload and hypoxic signals (12). These macrophages have excellent phagocytic ability and release immunoregulatory factors, such as IL-10 and TGF-β, at a higher rate depending on the local immune-regulatory condition (81). However, only correlations have been found thus far; direct regulatory effects on T-cell function require further verification.

Along with changes in macrophages, T cells in this area are also in a functionally limited activation state. CD8^+^ T cells continuously exposed to antigens and metabolic stress increase inhibitory receptors such as PD-1, TIM-3, and LAG-3, and their effector functions decrease (83). This state does not represent total immune system suppression. Studies have shown that CXCR6^+^ CD8^+^ T cells can be enriched in this area under the influence of IL-15 and contribute to hepatocyte injury (84). Local T cells still retain some effector functions, but they are not fully operational due to metabolic limitations. At the same time, homeostasis of Tregs is also compromised. Lipid-toxic environments repress mitochondrial fatty acid oxidation, reducing the expression and anti-inflammatory function of Foxp3 (85). This alteration may further impact local immune balance; however, its overall contribution to immune dysregulation requires quantitative evaluation. Based on spatial omics data, the immune cells in this area are generally undergoing a transition from oxidative phosphorylation to glycolysis and show some aging-associated traits (86). Overall, the local metabolic environment may alter the types of cells in the region and limit the activity of immune cells by adjusting their energy-metabolism pathway.

Therefore, zone 3 is best characterized as a functionally impaired immune microenvironment that has been modified by metabolic stress. The immune cells are not completely inhibited; rather, in this environment, they remain in a weakened state while simultaneously being subjected to metabolic pressure.

#### 3.1.3 Fibrotic septum

The fibrotic septum is a necessary structural component for the development of MASLD-associated bridging fibrosis and cirrhosis. It was previously believed that this structure served only to passively block the spread of inflammation. However, recent spatial omics studies have revealed that the immune features of this region are more complex. In research on MASLD and HCC, T cell infiltration has been found in the fibrotic region, with an overabundance of inhibitory receptors, revealing the immune status of the fibrotic septum in advanced-stage MASLD (80). At the same time, activated hepatic stellate cells (aHSCs) adjacent to the fibrotic septum release factors such as TGF-β and IL-10 to suppress lymphocyte function. A general feature of this area is a dissociation between the quantity and function of immune cells, that is, an increase in infiltration does not necessarily indicate an effective immune response, as the function of these cells is closely related to local microenvironmental signals. Extension of the extracellular matrix and modifications in tissue structure may inhibit the migration of immune cells and cell-to-cell communication. This spatial restriction mechanism may partially explain why immune checkpoint inhibitors have limited effectiveness against MASLD-associated HCC (15).

### 3.2 Spatial differentiation of immune cells

#### 3.2.1 KCs heterogeneity and replacement

Reduction of embryonically-derived resident KCs in MASLD is not uniform and shows strong spatial selectivity. KCs that express high levels of F4/80 and Tim4^+^ are more likely to be numerically reduced or functionally impaired in zone 3 (87). Based on these observations, KCs in different areas of the liver lobules likely have different levels of inherent resistance to metabolic stress; this difference may be due to extended hypoxia and increased lipid deposition in these areas. At steady state, bone marrow-derived monocytes and their differentiation into monocyte-derived KCs (MoKCs) in response to local microenvironmental signals help to maintain the stability of the macrophage population via monocyte recruitment to the liver (81). Local signals are integrated through Notch-RBPJ pathways and other metabolic or matrix-related factors during this process (88). However, there is no direct *in situ* evidence of spatial-specific changes in these regulatory mechanisms in MASLD. In the course of the disease, the injury-induced microenvironment at the periphery of zone 3 can change the differentiation pathway of monocytes, leading to the formation of short-lived inflammatory macrophages instead of the stable MoKC phenotype (81). Therefore, the local microenvironment influences how cells are recruited and what kind of cells they will be after differentiation. Changes in KCs of MASLD are better understood as a combination of “spatially non-uniform loss” and “limited *in situ* replacement” rather than a simple reduction in the number of cells.

#### 3.2.2 Macrophage differentiation trajectories

The differentiation of monocyte-derived macrophages in MASLD is spatially heterogeneous, and their diversity exceeds that of traditional M1/M2 classifications (12). Monocytes predominantly differentiate into Clec4e^+^ inflammatory macrophages in areas of fibrosis-associated inflammation and promote the spread of this inflammation (80). In specific metabolic or repair-related microenvironments, some cells are further differentiated into SPP1^+^/TREM2^+^ LAMs and are closely associated with lipid accumulation and local metabolic conditions. Lineage-tracing studies indicate that monocyte-derived macrophages undergo continuous differentiation, starting as Ly6C^hi monocytes, progressing through inflammatory intermediate stages, and finally becoming MoKCs (81). Therefore, this differentiation is continuous; these subpopulations of macrophages are not discrete but change constantly. It should be noted that, based on current data, it is generally believed that “the path of differentiation is affected by the local microenvironment”, but at present, it has not been determined whether spatial factors alone dictate cell differentiation fate. Locally produced lipids, Notch signaling pathway activation, and inflammatory factors all promote cell differentiation. As the disease progresses to the stage of HCC, the differentiation pathways of macrophages may diverge further, and some cells acquire tumor-associated characteristics (19). Histone lactylation represents another epigenetic mechanism that can maintain certain phenotypic states; thus, spatial microenvironmental signals may also affect the stability of cells by modifying epigenetics (29).

#### 3.2.3 T cell regional exhaustion and the REI

T cells are also distributed unevenly in space and show different functional states when exposed to MASLD. Existing studies have shown that CD8^+^ T cell exhaustion does not occur uniformly in the liver and is closely related to particular microenvironments. Spatial analysis shows that CD8^+^ T cells expressing inhibitory receptors such as PD-1 and TIM-3 are concentrated in the fibrotic septum area and overlap with cells that highly express TGF-β and IL-10. Based on these findings, it is hypothesized that the local cytokine environment sustains the state of exhaustion. These spatial characteristics are more prominent in MASLD-related HCC and are associated with differential responses to immunotherapy (89). However, the connection is currently based mainly on observations and does not provide direct evidence of causality.

Based on the above spatial features, the present review proposes a “REI” as a conceptual framework to quantify the functional status of T cells in different microanatomical regions, calculated as:


$$REI\;=\;\left(Density\;of\;PD-1^{+}TIM-3^{+}\;CD8^{+}\;T\;cells\;in\;a\;specific\;liver\;microanatomic\;region\right)\;÷\;\left(Total\;density\;of\;CD8^{+}\;T\;cells\;in\;that\;region\right)$$

This index is proposed to address the limitations of previous global analyses that did not consider spatial heterogeneity, thereby translating regional immune conditions into a quantifiable metric. The REI remains a conceptual framework at present, and its biological meaning and clinical utility have not been fully investigated. We acknowledge that this index requires validation through functional readouts, including IFNγ and GZMB production, TOX and LAG3 expression, Tcf1 expression, proliferative capacity, and spatial proximity analyses. Based on the available data, the REI is likely higher in zone 3 and the fibrotic septum area compared to zone 1, but further studies are needed to verify this. Hypoxia, lactate accumulation, and extracellular matrix deposition may all contribute to reduced T cell activity at the mechanistic level (15, 29). Future studies should evaluate whether REI correlates with clinical outcomes, such as fibrosis progression, treatment response, and survival, and should consider incorporating additional exhaustion markers and functional readouts to refine the index.

Oxygen and short-chain fatty acid concentrations decline progressively from the portal vein to the central vein, whereas lactate, free fatty acids, and hydrophobic bile acids increase in the opposite direction. Zone 1 is enriched with resident Kupffer cells and Tregs, where SCFAs may support Treg stability through epigenetic mechanisms. Zone 3 shows a different picture, with TREM2^+^ LAMs accumulating around the central vein, PD-1^+^TIM-3^+^ CD8^+^ T cells present in increased numbers, and a microenvironment characterized by hypoxia, lactate, and lipid overload. The fibrotic septum, when present, contains activated hepatic stellate cells, dense extracellular matrix, and immune cells with high inhibitory receptor expression. The REI, calculated as the ratio of PD-1^+^TIM-3^+^ CD8^+^ T cells to total CD8^+^ T cells in a given region, is proposed as a potential quantitative measure of regional T cell functional impairment, though it requires validation through functional readouts such as IFNγ, GZMB, TOX, LAG3, and Tcf1 expression, as well as proliferative capacity and spatial proximity analyses. Disease progression from early MASLD (left) to advanced MASH/cirrhosis (right) is shown along the horizontal axis.

### 3.3 Concluding summary and comparative immune markers across hepatic zones

The three hepatic zones exhibit distinct immune landscapes that evolve with disease progression. Zone 1 serves as the primary immune surveillance barrier, characterized by enrichment of resident KCs and Tregs, supported by SCFA gradients that maintain immune tolerance. In early MASLD, this zone loses its surveillance capacity due to reduced SCFA levels and impaired Treg function, allowing increased inflammatory signals to enter the liver. Zone 3 represents a metabolically stressed microenvironment with chronic hypoxia, lipid accumulation, and lactate excess. Here, LAMs accumulate, T cells show functional impairment with upregulated inhibitory receptors, and Treg stability is compromised by metabolic stress, collectively sustaining local inflammation and promoting fibrogenesis. The fibrotic septum, present in advanced disease, creates a structurally confined immunosuppressive niche where dense ECM and aHSCs physically and functionally inhibit immune cells, contributing to immune evasion and reduced immunotherapy efficacy. To facilitate comparison of these spatial immune features, **Table 1** summarizes the key immune cell populations, marker expression, and functional characteristics across the three zones.

**Table 1:** Comparative immune characteristics of hepatic zones in MASLD.

Feature	Zone 1 (Periportal)	Zone 3 (Pericentral)	Fibrotic Septum
Oxygen tension	Relatively high	Low (hypoxic)	Severely hypoxic
Key metabolites	SCFAs, primary bile acids	Lactate, free fatty acids, hydrophobic bile acids	Lactic acid, ECM components
Dominant macrophages	Resident KCs (Tim4^+^F4/80^+^), Ly6C^lo patrolling monocytes	TREM2^+^ LAMs, DTNA^+^ macrophages	Infiltrating macrophages, aHSC-associated
T cell profile	Treg-enriched, naïve T cells	CD8^+^ T cells with PD-1^+^TIM-3^+^, reduced effector function	High-density PD-1^+^TIM-3^+^LAG-3^+^ T cells
Treg status	Stable, SCFA-supported	Metabolically compromised, reduced Foxp3	Functionally suppressed by TGF-β/IL-10
Key functional state	Immune surveillance/tolerance	Metabolic inflammation, functional impairment	Structural immune confinement
Disease stage prominence	Early MASLD	MASH, early fibrosis	Advanced fibrosis (F3-F4), cirrhosis

## 4 Spatial metabolism-immune interactions

The spatial immune landscape described in the preceding chapter is not a static entity; rather, it is dynamically orchestrated by regionally distinct metabolic signals. Herein, we examine how metabolites function as signaling cues to modulate immune cell responses in a zone-dependent manner. The MASLD liver exhibits a perturbed metabolic gradient along the portal-central axis, with lipids, SCFAs, bile acids, and TCA cycle intermediates showing differential spatial distribution (11). These metabolites directly modulate immune cell function through receptor-mediated signaling, epigenetic modifications, and metabolic competition. We first discuss lipid partitioning as a key driver of immune regulation, then examine specific metabolite signaling axes, and finally consider the metabolic vulnerability of Tregs and nutritional competition within immune-metabolic niches. Throughout, we emphasize the spatial association between metabolite gradients and immune cell phenotypes, highlighting how the metabolic microenvironment reinforces regional immune dysfunction.

### 4.1 Lipid partitioning as a key driver of immune regulation

#### 4.1.1 Spatial association between cholesterol deposition and inflammasome activation

Disorders of cholesterol metabolism are believed to contribute to the progression from MASLD to MASH. Studies have demonstrated that free cholesterol deposition in the liver is spatially biased, predominantly accumulating in hepatocytes and macrophages surrounding the central vein. Excessive intracellular cholesterol accumulation can form crystalline structures and has been shown to activate the NLRP3 inflammasome, thereby promoting the maturation and release of inflammatory factors such as IL-1β (90). Spatial omics and multiplex immunofluorescence analyses further revealed that IL-1β-positive macrophages and aHSCs co-localize around the central vein and at the edges of the fibrotic septa (80). These findings suggest a spatial association among cholesterol deposition, inflammasome activation, and fibrotic cells.

#### 4.1.2 Short-chain fatty acid gradients and regional support of Tregs

SCFAs, such as butyric acid and propionic acid, are produced by the fermentation of gut microbiota and enter the liver through the portal vein, creating a concentration gradient that decreases from the portal vein to the central vein. SCFAs can inhibit HDACs and promote Foxp3-related epigenetic modifications to maintain the stability and function of Tregs (91). Dysbiosis of the gut microbiota in MASLD reduces the production of SCFAs (92, 93). Spatially, this global reduction may lead to an even more prominent decrease in SCFA concentration in the vicinity of the portal vein and thus disrupt the original gradient structure. Although there is an established association between SCFA reduction and impaired immune regulatory function, direct evidence quantifying changes in Treg populations across different regions of the liver lobule and their spatial dependence remains lacking. Therefore, a more cautious interpretation is that alterations in the SCFA gradient may contribute to the regulation of liver immune homeostasis and, to some extent, influence the inflammatory response in portal vein-adjacent regions.

#### 4.1.3 Oxidized lipids and LAM differentiation

The accumulation of oxidized lipids in zone 3 is closely associated with the differentiation state of monocyte-derived macrophages. Studies have demonstrated that the formation of TREM2^+^ LAMs correlates with the local lipid load (12). Spatial transcriptomics analysis has revealed that TREM2^+^ macrophages are enriched in zone 3 and the fibrotic septum region, where they co-localize with hypoxia- and fibrosis-related signals (41). This distribution pattern suggests that the local metabolic environment may play a role in regulating the spatial differentiation of macrophages. Functionally, LAMs exhibit robust lipid-processing capabilities and secrete immunoregulatory factors such as IL-10 and TGF-β; their presence is associated with the local immune regulatory state. Furthermore, oxidized lipids can influence macrophage phenotype by activating metabolism-related pathways, including PPAR-γ. Current research indicates that these signals may be more active in zone 3, thereby exacerbating spatial differences in macrophage distribution and function.

### 4.2 Metabolite signaling axes

#### 4.2.1 Succinate and monocyte recruitment

Lipotoxicity and mitochondrial dysfunction can lead to the accumulation of tricarboxylic acid (TCA) cycle intermediates, particularly succinate, within hepatocytes and their subsequent release into the extracellular space. Succinate binds to the receptor SUCNR1 (GPR91) on monocytes and dendritic cells and promotes the recruitment of inflammatory cells, thereby exacerbating inflammation. The more metabolically impaired the areas of MASLD, the more mitochondrial dysfunction they are likely to have. According to recent studies, these areas are expected to be relatively high-yield sources of succinate release. However, no direct spatial metabolomics studies have demonstrated the formation of a stable chemotactic gradient of succinate in the hepatic lobule. Succinate can also increase the rate of glycolysis and the expression of inflammatory genes by promoting HIF-1α stabilization, thereby sustaining a pro-inflammatory state in macrophages. Although this is not its primary function in cell positioning, certain areas have shown local responses involving immune cells over time.

#### 4.2.2 Bile acid signaling and KC function

Bile acids (BAs) are key immunomodulators in the gut-liver axis that act through the FXR and TGR5 receptors. There is a spatial gradient in the distribution of primary and secondary BAs in the liver; newly synthesized BAs are located near the portal vein, and more hydrophobic BAs are distributed along the direction of blood flow. FXR maintains the anti-inflammatory and barrier-protective functions of KCs, and TGR5 activates the cAMP pathway in KCs to suppress LPS-induced inflammatory factors strongly (28, 88). Hydrophobic bile acids in the blood and liver are more abundant in MASLD and correlate with the extent of the disease (94). Spatiotemporally, this leads to an uneven distribution of FXR/TGR5 signaling in KCs close to the portal vein: reduced intermittent activation of FXR weakens its anti-inflammatory function at the barrier, and overstimulation of TGR5 may result in immunosuppression, thereby hindering the clearance of intestinal PAMPs by KCs. Thus, the mechanism may help explain why MASLD patients are more susceptible to sepsis. Recently, allo-LCA and other new bile acids have been found to reduce liver fibrosis by targeting the TGR5 pathway, offering new directions for treatment (95).

### 4.3 Metabolic vulnerability of Tregs

Metabolic sensing pathways are not distributed evenly across different areas of the liver lobule, and all are essential components in regulating the immune system (96). LXRα, a hub for lipid and immune regulation, is now known to exist in many parts of the body under different metabolic conditions (97). LXRα is activated in KCs in zone 1 to promote cholesterol excretion and the transcription of anti-inflammatory genes. On the other hand, hepatocytes in zone 3, having suffered from long-term fat accumulation, continuously express LXRα; its activation promotes SREBP-1c-mediated synthesis of lipids and thus increases the local lipotoxic environment. Direct *in situ* evidence of the functional differentiation of LXRα across spatial regions is still lacking; thus far, only cell type and metabolic state differences have been indirectly identified in terms of spatial distribution. Tregs are relatively sensitive to metabolic alterations and require ATP provided by fatty acid oxidation in the mitochondria for survival. Research has shown that, in a lipotoxic state, long-chain saturated fatty acids and oxidized lipids suppress mitochondrial function and promote the instability of Foxp3 (7). Spatial metabolomics studies also show that zone 3 is relatively enriched in lipotoxic metabolites, such as long-chain acylcarnitines and ceramides, which are associated with changes in mitochondrial permeability and cell death. Based on the above evidence, it is reasonable to hypothesize that the stability of Tregs differs among the regions of hepatic lobules, and areas with higher lipid loads may be more susceptible to metabolic stress.

### 4.4 Nutritional competition and immune-metabolic niches

Different types of immune cells have different metabolic bases for their function (98). Effector T cells are generally glycolytic and thus have a relatively high capacity for energy production; on the other hand, Tregs are more dependent on fatty acid oxidation for energy supply and are employed to suppress the immune system (99, 100). Different cell populations have different metabolic requirements and are thus differentially affected by the metabolically restricted environment. In MASLD, zone 3 is frequently hypoxic and fatty, and thus the local environment is altered. Single-cell analysis shows that CD8^+^ T cells in this area have increased expression of glucose transporter 1 and lactate dehydrogenase A, indicating enhanced glycolysis, while Tregs show reduced expression of fatty acid oxidation-related genes (101). Therefore, different types of immune cells exhibit varied metabolic adaptations in the same microenvironment. Furthermore, myeloid-derived suppressor cells are enriched in MASLD, particularly in fibrotic regions, where they inhibit T cell function by regulating arginine metabolism and producing nitric oxide, thereby disrupting the local immune metabolic environment (102).

## 5 Spatial immune dysregulation and disease progression

Chemokine imbalance, modification of the metabolic microenvironment, desynchronization of circadian rhythms, and specific metabolic-immune interactions do not occur uniformly in the liver lobule (103). They are selectively filtered and enhanced by the inherent metabolic polarity of the liver lobules. Finally, all of these disturbances converge at three main spatial locations: zone 1, zone 3, and the fibrotic septum. Consequently, a series of immunopathological processes occur sequentially: reduced immune surveillance, exhaustion of effector functions, and finally, structural immune confinement. These three modules operate in series and form a vicious circle that generates a spatial immune-driving force for the development of simple steatosis, cirrhosis, and even HCC (104, 105).

### 5.1 Reduced immune surveillance capacity

Zone 1 is near the entrance of blood vessels supplying the liver from the intestine and serves as an important link that transmits signals from the intestine to the immune system in the liver. Under steady-state conditions, this region is enriched with embryonically derived KCs, which continuously clear low levels of intestinal PAMPs via pattern recognition receptors, thereby maintaining a low-inflammatory state characterized by immune tolerance (79). This steady state is not the result of a single-cell function but rather depends on the synergistic action of local metabolic signals and immune regulatory networks. Among these, SCFAs constitute an important metabolic regulatory axis in this region. SCFAs, produced by the fermentation of dietary fibers by gut microbiota, enter the liver through the portal vein and reach relatively high concentrations in zone 1. Studies have shown that SCFAs can inhibit HDAC activity, enhance Foxp3-related epigenetic modifications, and thereby support the stability and immunosuppressive function of Tregs (91). Therefore, the SCFA gradient may play a supportive role in maintaining the immune tolerance state of zone 1. In the early stages of MASLD, this area first exhibits disturbances in immune homeostasis; these changes are not limited to increased inflammation but also include reduced immune-regulatory function and increased susceptibility to inflammation. Spatial transcriptomics research shows that before serious fibrosis occurs, some chemokines in zone 1 have already changed; for example, *Cxcl9* expression has dropped, impairing the recruitment of effector T cells (80). Therefore, this area may be the first to show reduced immune surveillance capacity.

At the same time, dysbiosis of the gut microbiota associated with MASLD reduces the production of SCFAs and thus lowers SCFA levels in portal vein blood (92). This reduction may be more pronounced in zone 1 because this area is accustomed to higher SCFA concentrations for immune regulation. Although there is no direct *in situ* evidence showing that reduced in SCFAs causes a local decline in the number or function of Tregs, several studies have supported a functional connection between SCFAs and Treg stability. Therefore, reduced Treg function may decrease the inhibitory effect they have on KCs and monocyte-derived macrophages, thus lowering the activation threshold for intestinal PAMPs. Research has found that, in MASLD, KCs can show a pro-inflammatory phenotype and increase the levels of TNF-α and IL-1β (7). At the same time, recruitment of Ly6C^hi inflammatory monocytes is increased, indicating enhanced local inflammatory signals. A disorder in the multiple domains of the microbiome (bacteria, viruses, fungi) can reduce bile acid metabolism and thus alter the immune status of this area (25). These factors may lead to the conversion of zone 1 from an immune-tolerant area to an inflamed one. In summary, in the early stages of MASLD, zone 1 shows a reduction in immune-regulating ability and a lowered threshold for inflammation, possibly due to SCFA deficiency, defective Treg function, activation of KCs, and other factors.

### 5.2 Zone 3: functional impairment and chronic inflammation

Zone 3 has a relatively high metabolic demand within the hepatic lobules and is chronically exposed to a microenvironment with relatively high concentrations of free fatty acids, hypoxia, and lactate. Therefore, this metabolic profile generates a particular environment for the function of local immune cells, and the resulting immune phenotype is neither a simple expansion of inflammation nor an effective response, but rather a complex state of both immune activation and functional deficiency.

#### 5.2.1 Spatial enrichment and functional characteristics of LAMs

In zone 3, infiltrating monocytes differentiate into TREM2^+^ LAMs under the influence of lipid accumulation and hypoxic signals. Lineage tracing studies have shown that they are mainly derived from peripheral monocytes and not from embryonic resident KCs (12). Spatial multi-omics analysis has also revealed that LAMs are enriched in zone 3 and fibrosis-related regions, and they are close to aHSCs (80). Based on cell communication analysis, LAMs may be involved in the activation of HSCs through specific ligand-receptor pathways, such as the RUNX2-PLG-PARD3 axis (41). Functionally, LAMs are efficient phagocytic cells that also produce immunomodulatory factors such as IL-10 and TGF-β (81). LAMs are thus expected to promote the formation of an immunomodulatory microenvironment rather than simply an immunosuppressive structure. However, it should be noted that the functional roles of LAMs remain controversial; while some studies suggest a hepatoprotective role through HGF secretion (11), others emphasize their pro-fibrotic potential via HSC activation. The net contribution of LAMs to MASLD progression may depend on disease stage, spatial context, and the balance between these opposing functions.

#### 5.2.2 Spatial characteristics of functionally limited T cells

CD8^+^ T cells in zone 3 are not significantly reduced in number, but they are functionally impaired and express inhibitory receptors such as PD-1, TIM-3, and LAG-3, along with reduced production of effector cytokines including IFN-γ and TNF-α (83). Multiple metabolic factors likely contribute to this state. Hypoxia alters the metabolic program of T cells through HIF-1α, stimulates activation of the glycolytic pathway, and locally accumulated lactate may also modulate immune cell function epigenetically (29). In addition, CXCR6^+^ CD8^+^ T cells can also be recruited to zone 3 and fibrotic areas under IL-15-induced conditions, causing damage to hepatocytes (84). Notably, these cells also express exhaustion-related markers and are in a state of continuous activation with impaired function. Overall, the T cells in zone 3 are closer to a state of “spatially limited functional impairment” than to complete immune failure. The above conditions may result from metabolic stress, antigen stimulation, or other factors.

#### 5.2.3 Spatial dependence of Treg functional stability

Tregs in zone 3 are also subject to the same metabolic stress as effector T cells. Tregs require mitochondrial fatty acid oxidation to function normally. Long-chain fatty acids and oxidized lipids in zone 3 can interfere with this metabolic process and reduce the stability of Foxp3 expression (85). Based on the above, it is inferred that in zone 3, the loss of Treg function is due to metabolic suppression. Zone 1 lacks Treg support due to decreased SCFAs, but zone 3 is different in that a harmful metabolic environment locally suppresses Treg function. As a result, the metabolic environment in zone 3 may reduce the immune regulatory function of Tregs and lead to loss of local immune homeostasis.

### 5.3 Fibrotic septum: structural immune confinement

When the disease reaches the F3–F4 stage, a new spatial structural unit of the liver has formed through fibrosis. For a long time, it was thought that this structure served only to passively block the spread of inflammation. However, recent spatial omics research has shown that this is not the case; rather, the fibrotic septum can be considered both a spatially and metabolically confined site for immune functions. This area shows a paradoxical situation of high-density immune cell infiltration accompanied by strong functional suppression. The infiltrating T cells and B cells highly express inhibitory receptors such as PD-1, TIM-3, and LAG-3, and also show an abnormal increase in immunoglobulin genes and senescence-associated secretory phenotype (SASP) characteristics (80). Dense extracellular matrix (ECM) deposition physically obstructs contact between T cells and target cells; simultaneously, activated mechanosensors such as Piezo1 release chemokines and adhesion molecules continuously, disrupting the normal spatial navigation signals of immune cells (76). Under severe hypoxia and lactic acidosis near the septum, a highly anaerobic microhabitat has formed; concurrently, epigenetic alterations such as histone lactylation occur to maintain the anti-regulatory phenotype of T cells (29). More importantly, the active core component of the septum is aHSCs, which continuously secrete TGF-β and IL-10 to maintain and enhance this inhibitory microenvironment. Therefore, the fibrotic septum shows a typical feature: although there is infiltration of immune cells in this area, they are structurally and functionally inhibited, resulting in dissociation from the process of inflammation. This may partially explain the relatively high failure rate of immunotherapy in MASLD-related HCC.

### 5.4 Integrated model of spatial immune-driven progression

The spatial immune alterations across these three zones form an integrated model of disease progression. In early MASLD, zone 1 loses its immune surveillance capacity due to reduced SCFA gradients and impaired Treg function, allowing increased inflammatory signals to enter the liver. As disease advances to MASH, zone 3 becomes a site of chronic metabolic stress, characterized by LAM accumulation, T cell functional impairment, and Treg instability, which together sustain local inflammation and promote fibrogenesis. In advanced stages (F3-F4 cirrhosis), the fibrotic septum creates a structurally and metabolically confined microenvironment that physically and functionally inhibits immune cells, contributing to immune evasion and reduced immunotherapy efficacy. This sequential, spatially defined progression, from immune surveillance failure in zone 1, to functional impairment in zone 3, to structural confinement in the fibrotic septum, represents a conceptual framework for understanding MASLD pathogenesis and may guide the development of zone-specific therapeutic strategies. However, direct longitudinal evidence confirming this temporal sequence is currently lacking; prospective studies tracking spatial immune changes over time are urgently needed to validate this model (**Table 2**).

**Table 2:** Summary of key spatial omics and single-cell studies in MASLD/MASH.

Sample types	Disease stages	Platforms, major cell populations	Spatial regions	Principal findings	Reference
Human liver biopsies (n=61: 10 CTRL, 17 MASL, 34 MASH)	CTRL, MASL, MASH (F1-F4)	scRNA-seq (540,216 cells), 10x Visium spatial transcriptomics (47,864 spots), MALDI-MSI spatial metabolomics, spatial proteomics; LAMs, KCs, HSCs, ECs, immune cells	Periportal, pericentral, fibrotic regions	• Identified MITF as key regulator of LAM lipid-handling capacity via PGC1α-PPARγ-FAO axis • Revealed hepatoprotective role of LAMs via HGF secretion • Delineated fibrosis-associated gene program enriched in advanced MASH • Identified RSPO3-LGR6 crosstalk between central vein ECs and HSCs in fibrotic regions	(11)
Human liver FNAs and paired PBMCs (n=25: 2 NS, 4 SS, 5 F0, 5 F1, 5 F2, 4 F3/F4)	NS-SS, early MASH (F0-F1), advanced MASH (F2-F4)	scRNA-seq (168,634 cells); T cells, NK cells, B cells, monocytes, macrophages, DCs	Whole liver	• Heightened immunoregulatory programs with MASH progression: enriched Treg cells, M-MDSCs, TREM2^+^S100A9^+^ macrophages, S100^hiHLA^lo cDC2s • Hepatic cytotoxic T cell functions increased with inflammation but decreased with fibrosis, acquiring exhausted signature • NK cell-driven toxicity intensified with fibrosis progression	(8)
Human and mouse liver (115 scRNA-seq samples, 7 spatial samples)	Healthy, MASLD, MASH (eMASH F0-2, aMASH F3-4)	scRNA-seq (570,194 cells), spatial transcriptomics (7 MASH samples), bulk RNA-seq, ATAC-seq; hepatocytes, ECs, T cells, monocytes/macrophages, HSCs, cholangiocytes, B cells	Spatial co-localization analysis; periportal/pericentral regions	• Identified DTNA^+^ macrophages specifically enriched in MASH with M2 polarization and hypoxia/HIF activation • RUNX2 identified as key transcriptional regulator of DTNA^+^ macrophages • DTNA^+^ macrophages interact with aHSCs via RUNX2-PLG-PARD3 axis • Ensemble machine learning identified DTNA as optimal predictive biomarker (mean AUC = 0.839)	(41)
Human PBMCs (n=36: 22 MASLD/MASH, 14 HC)	MASLD/MASH (fibrosis < F3, stratified by NAS: low <3, medium 3-4, high >4)	scRNA-seq (29,890 cells), bulk RNA-seq (liver and WBC); neutrophils, monocytes, B cells, T cells	Peripheral blood	• Increased abundance of immature B cells in patients with MASLD/MASH • Increased abundance of myeloid-derived suppressor cells (MDSC) in patients • Immature circulating neutrophils associated with MASLD • Enrichment of transitional B cells in MASLD • Depletion of naive-like CD4^+^ T cells and enrichment of Tregs in MASLD	(80)

The literature search was conducted in PubMed and Web of Science databases using the following search terms and their Boolean combinations: “MASLD,” “MASH,” “metabolic dysfunction-associated steatotic liver disease,” “spatial transcriptomics,” “single-cell RNA sequencing,” “liver lobule,” “immune,” “immune cell,” “macrophage,” and “hepatic stellate cell.” The search was restricted to peer-reviewed articles published between January 2021 and June 2026. Additional relevant studies were identified by manual screening of reference lists of retrieved articles and key reviews.

## 6 Clinical implications and future perspectives

### 6.1 Biomarker development

The spatial immune landscape of MASLD offers opportunities for biomarker discovery. Regional differences in immune cell composition, particularly the enrichment of LAMs in zone 3 and the presence of exhausted T cells in the fibrotic septum, may be detectable through minimally invasive approaches. Emerging liquid biopsy technologies, including analysis of circulating immune cell populations, cell-free DNA methylation patterns, and extracellular vesicle cargo, may reflect spatial immune alterations in the liver (106). However, the correlation between peripheral blood markers and regional liver immune status requires validation in large cohort studies. The REI, once validated, could potentially serve as a tissue-based biomarker to assess regional T cell dysfunction and may predict disease progression or treatment response, although this remains to be established in prospective cohorts.

### 6.2 Patient stratification

MASLD is increasingly recognized as a heterogeneous disease with distinct clinical subtypes (107). Spatial immune profiling may, in the future, enable more precise patient stratification by identifying individuals with predominant zone 1 dysfunction (loss of immune tolerance), zone 3 metabolic inflammation (LAM-driven fibrosis), or fibrotic septum immune confinement (immunotherapy resistance). Such stratification could guide clinical trial design and personalized treatment selection. For example, patients with high REI in the fibrotic septum might be candidates for combination immunotherapy approaches targeting both immune checkpoints and the fibrotic microenvironment, whereas those with predominant zone 1 alterations might benefit from gut microbiome modulation or SCFA supplementation.

### 6.3 Spatially targeted therapeutic strategies

The spatial framework outlined in this review suggests potential future directions for therapeutic intervention that move beyond conventional systemic approaches. Unlike traditional strategies that treat the liver as a homogeneous organ, spatially targeted therapies would aim to correct region-specific immune dysfunctions by exploiting the distinct metabolic and structural characteristics of each zone.

Metabolic microenvironment remodeling represents one promising direction for restoring immune function in zone 3, where chronic hypoxia, lactate accumulation, and lipid overload impair immune cell activity. Pharmacological inhibition of HIF-1α has shown preclinical promise in reducing NLRP3 inflammasome activation and liver fibrosis (44), while interventions that lower lactate or block histone lactylation may reverse pro-inflammatory macrophage phenotypes (29). Chronobiological interventions such as time-restricted feeding offer non-pharmacological means to restore rhythmic immune function (69, 77).

Zone-specific drug delivery capitalizes on nanoparticle engineering to achieve preferential accumulation in defined liver regions. Portal vein administration preferentially reaches zone 1, whereas strategies designed to penetrate the fibrotic septum could overcome physical barriers in advanced disease (15).

Combination approaches may eventually be needed given the spatial heterogeneity of immune dysfunction, but the optimal combination and sequencing remain undefined. The frequent failure of immune checkpoint therapy pertains mainly to the HCC context and should not be directly extrapolated to MASLD itself. While the spatial framework provides a rational basis for designing next-generation therapeutic regimens, translation to clinical practice faces substantial hurdles, including the complexity of trial design, potential synergistic toxicities, and the lack of validated biomarkers to guide combination selection.

## 7 Conclusion and future directions

Research on the immunopathological mechanism of MASLD has gradually moved away from traditional cell counting and phenotypic polarization methods toward a multi-scale analysis system that incorporates spatial information. Spatial transcriptomics and multiplex immunofluorescence have shown that immune cells are not distributed randomly in the hepatic lobules but form functionally distinct regional microenvironments in zone 1, zone 3, and the fibrotic septum. Enrichment of LAMs in zone 3 (12), spatial co-localization of DTNA^+^ macrophages with aHSCs (41), and functionally restricted state of CD8^+^ T cells in the fibrotic septum together constitute the main evidence for spatial immune dysregulation (106).

### 7.1 Methodological gaps and future technologies

Several methodological challenges remain in building a spatial immunology framework. First, current spatial transcriptomics technologies have limited resolution for metabolite detection, hindering integrated analysis of metabolic gradients and immune cell functional states. Developing a high-resolution “spatial metabolome–immunome” multi-omics detection platform will be essential to verify the hypothesis that the metabolic microenvironment reshapes the spatial behavior of immune cells. To move from correlational observations to causal inference, future research should employ zonal targeted gene editing or cell-type-specific ablation in animal models to functionally validate the role of specific immune populations in distinct lobular regions. For instance, conditional knockout of HIF-1α specifically in zone 3 hepatocytes or macrophages could determine whether hypoxia-driven signals are causally linked to LAM accumulation and fibrosis. Similarly, region-specific restoration of SCFA signaling in zone 1 could test whether Treg stability is directly dependent on local metabolic cues. These approaches, combined with *in vivo* imaging, would enable targeted interventions in zone 1 and zone 3, respectively, to clarify the specific role of spatial immune dysregulation in disease progression. Second, there is currently a lack of animal models capable of functional interventions within specific hepatic lobules, limiting the ability to move from correlation to causal inference. Third, prospective longitudinal studies in human cohorts are needed to establish the temporal sequence of spatial immune alterations and their relationship to clinical outcomes. Current cross-sectional data cannot distinguish whether spatial immune changes are drivers or consequences of disease progression. Fourth, the reproducibility of spatial findings across different patient populations and technical platforms must be systematically evaluated. Addressing these gaps will be critical for translating spatial immune concepts into clinical practice.

### 7.2 Unanswered questions

Several key questions remain unanswered and warrant future investigation. First, what is the temporal sequence of spatial immune alterations during MASLD progression? Does zone 1 dysfunction precede zone 3 inflammation, or do these processes occur in parallel? Second, what is the relative contribution of each driving mechanism (chemokine remodeling, metabolic reprogramming, circadian disruption) to spatial immune dysregulation? Third, do different clinical subtypes of MASLD exhibit distinct spatial immune patterns? Fourth, how do sex differences and genetic susceptibility influence spatial immune organization? Fifth, what is the role of extracellular vesicles in mediating spatial immune signaling between zones? Addressing these questions will require longitudinal studies, large cohort analyses, and integration of multi-omics data.

### 7.3 Therapeutic implications and outlook

Although still in the early stages of development, the spatial immunology perspective offers new conceptual insights for designing MASLD intervention strategies. On one hand, regulating the chemokine network or interfering with rhythm-related factors may influence the spatial distribution patterns of immune cells to some extent (108). On the other hand, exploring strategies for “metabolic microenvironment remodeling” holds theoretical potential for overcoming the limitations imposed by the local metabolic microenvironment on immune cell function; however, related research remains at the conceptual exploration stage.

As an exploratory indicator proposed in this paper, the REI is intended as a conceptual framework, rather than a validated biomarker, for describing regional differences in T cell functional status. Its clinical predictive value and reproducibility require further validation in independent cohorts, and its utility as a clinical tool remains to be established. Future studies should evaluate whether REI correlates with fibrosis progression, treatment response, and survival, and should consider incorporating additional markers and functional readouts to refine the index, including IFNγ, GZMB, TOX, LAG3, Tcf1 expression, proliferative capacity, and spatial proximity analyses.

More broadly, the transition from correlation to causation represents a major challenge for the field. While spatial omics have provided rich descriptive data, experimental validation of causal relationships through zonal gene editing, pharmacological perturbations, and longitudinal intervention studies is urgently needed. Furthermore, the clinical translation of spatial immune concepts will require the development of standardized assays, reference ranges, and multicenter validation cohorts. Despite these challenges, the spatial perspective offers a useful complement to existing paradigms and may ultimately inform the development of spatially targeted therapeutic strategies tailored to the distinct immune environments of each hepatic zone.

In conclusion, the spatial perspective on immune dysregulation in MASLD offers a useful complement to existing paradigms and may inform the development of spatially targeted therapeutic strategies. As spatial omics technologies continue to advance, the integration of spatial, metabolic, and immune data will likely provide deeper insights into MASLD pathogenesis and may eventually enable more precise, personalized approaches to diagnosis and treatment. However, the transition from correlative observations to causally validated mechanisms and clinically applicable tools will require sustained efforts in method development, mechanistic investigation, and prospective clinical validation.
